# Seasonal patterns of sickness absence due to diagnosed mental disorders: a nationwide 12-year register linkage study

**DOI:** 10.1017/S2045796023000768

**Published:** 2023-11-09

**Authors:** M. Virtanen, S. Törmälehto, T. Partonen, M. Elovainio, R. Ruuhela, C. Hakulinen, K. Komulainen, J. Airaksinen, A. Väänänen, A. Koskinen, R. Sund

**Affiliations:** 1 School of Educational Sciences and Psychology, University of Eastern Finland, P.O. Box 111, Joensuu, Finland; 2 Division of Insurance Medicine, Department of Clinical Neuroscience, Karolinska Institutet, 17177 Stockholm, Sweden; 3 Department of Public Health and Welfare, Finnish Institute for Health and Welfare, P.O. Box 30, 00271 Helsinki, Finland; 4 Department of Psychology and Logopedics, University of Helsinki, P.O. Box 4, 00014 Helsinki, Finland; 5 Research Program Unit, Faculty of Medicine, University of Helsinki, P.O. Box 4, 00014 Helsinki, Finland; 6 Weather and Climate Change Impact Research, Finnish Meteorological Institute, P.O. Box 503, 00101 Helsinki, Finland; 7 Finnish Institute of Occupational Health, P.O. Box 40, 00032, Helsinki, Finland; 8 Institute of Clinical Medicine, University of Eastern Finland, P.O. Box 1627, 70211 Kuopio, Finland

## Abstract

**Background:**

Although seasonality has been documented for mental disorders, it is unknown whether similar patterns can be observed in employee sickness absence from work due to a wide range of mental disorders with different severity levels, and to what extent the rate of change in light exposure plays a role. To address these limitations, we used daily based sickness absence records to examine seasonal patterns in employee sickness absence due to mental disorders.

**Method:**

We used nationwide diagnosis-specific psychiatric sickness absence claims data from 2006 to 2017 for adult individuals aged 16 to 67 (n=636,543 sickness absence episodes) in Finland, a high-latitude country with a profound variation in daylength. The smoothed time-series of the ratio of observed and expected (O/E) daily counts of episodes were estimated, adjusted for variation in all-cause sickness absence rates during the year.

**Results:**

Unipolar depressive disorders peaked in October-November and dipped in July, with similar associations in all forms of depression. Also anxiety and non-organic sleep disorders peaked in October-November. Anxiety disorders dipped in January-February and in July-August, while non-organic sleep disorders dipped in April-August. Manic episodes reached a peak from March to July and dipped in September-November and in January-February. Seasonality was not dependent on the severity of the depressive disorder.

**Conclusions:**

These results suggest a seasonal variation in sickness absence due to common mental disorders and bipolar disorder, with high peaks in depressive, anxiety, and sleep disorders towards the end of the year and a peak in manic episodes starting in spring. Rapid changes in light exposure may contribute to sickness absence due to bipolar disorder. The findings can help clinicians and workplaces prepare for seasonal variations in healthcare needs.

Seasonal variation in mental disorders is a well-known phenomenon and it has been suggested to be linked to a failure in the harmonization of circadian rhythms and external day-night rhythm, in which changes in light exposure play a central role (LeGates *et al.;* 2014, Zhang and Volkow, 2023). The observed higher prevalence of mental disorders in high-latitude compared to low-latitude regions is associated with malfunctions in the biological adaption to prominent light changes in high-latitude areas, which deviate strongly from the humans’ original environment close to the equator (Rosenthal *et al.;* 2020, Zhang and Volkow, 2023).

Seasonal variation is not similar in all mental disorders and within disorders, the findings have been mixed. However, some consistencies have been observed. Unipolar depressive episodes seem to be most prevalent in winter and least prevalent in the summer months (Gardarsdottir *et al*., 2010; Patten *et al*., 2017; Overland *et al*., 2019; Bakstein *et al*., 2020; Fellinger *et al*., 2022). However, previous research, including a systematic review, has suggested that the winter peak is more likely in clinical depression than in milder forms of depression (LoBello and Mehta, 2019; Overland *et al*., 2019). Seasonal affective disorder (SAD) is a subtype of recurrent depressive disorder or bipolar disorder, with symptoms of depressive mood, lack of energy, hypersomnia and increased appetite usually beginning in the autumn and remitting in the spring (Rosenthal *et al*., 1984; Partonen and Lonnqvist, 1998). In bipolar disorder, manic episodes seem to peak during the spring or summer and, to a lesser extent, in the autumn, whereas depressive episodes mostly peak in early winter, and mixed episodes peak in early spring as well as in the middle of or late in the summer (Geoffroy *et al*., 2014; Fellinger *et al*., 2022; Zhang and Volkow, 2023). Few studies have focused on anxiety, sleep and substance use disorders, although these have been associated with self-reported SAD (Sandman *et al*., 2016; Palmu et al., 2022).

Mental disorders are associated with substantially impaired workforce participation, such as sickness absence due to illness, and to date, they are the most common cause in the nationally recorded sickness absence in Finland, affecting 100,000 individuals annually (Social Insurance Institution of Finland, 2023). Previous research has recognized several risk factors of sickness absence due to mental disorders, such as working conditions (Duchaine *et al*., 2020) and lifestyle factors (Virtanen *et al*., 2018), but little attention has been paid to seasonality. Seasonal trends in sickness absence can provide important information on the burden of mental disorders and provide answers to questions such as how the rates vary during the year, and how healthcare services and organizations could prepare their functions accordingly. Furthermore, it is unknown whether seasonal variation in sickness absence corroborates with the previously observed seasonality of psychiatric morbidity measured by hospital admissions and healthcare use (Geoffroy *et al*., 2014; Dominiak *et al*., 2015; Overland *et al*., 2019; Zhang *et al*., 2021; Törmälehto *et al*., 2022; Fellinger *et al*., 2022; Rosenthal *et al*., 2020) as well as survey- and interview-based data (Winthorst *et al*., 2011; Friborg *et al*., 2012; Johnsen *et al*., 2012; Geoffroy *et al*., 2014; Patten *et al*., 2017; Putilov, 2017; Overland *et al*., 2019; LoBello and Mehta, 2019; Sivertsen *et al*., 2021). Conditions that require hospital treatment are at the most severe end of the disease continuum. Only one previous study has examined seasonality in sickness absence among general practice patients who started antidepressant treatment during the winter, and it found that sickness absence rates peaked at the time of antidepressant initiation (Winkler *et al*., 2019).

We used nationwide register data to investigate seasonal patterns in sickness absence due to mental disorders among the general population of Finland, a high-latitude country with a profound variation in daylength (photoperiod). The specific aims of this study were: 1) to examine the seasonal patterns of sickness absence in a variety of mental disorders; 2) to assess whether seasonality is similar in milder and more severe forms of depression; and 3) to investigate whether the rate of change in light exposure, as suggested by Rosenthal *et al*. (2020, 2021), is associated with changes in sickness absence rates.

## Material and methods

### Study population

We obtained daily data on sickness absence from the nationwide sickness absence claims register of The Social Insurance Institution of Finland (Kela), from Jan 1 2006 to Dec 31 2017, covering 12 years (Hakulinen *et al*., 2020). All individuals aged 16 to 67 with a medically certified work disability are eligible for sickness allowance, irrespective of their employment status. The waiting period is the first day of the illness based on a medical certificate and the subsequent nine working days. The employer is obliged to pay a salary and an unemployed person receives unemployment benefit during the waiting period. The anonymized sickness absence data include the main diagnosis (the reason for work disability) and the starting and ending days of each absence period, including the waiting periods. The main diagnosis for each sickness absence period was coded according to the International Classification of Diseases 10^th^ edition (ICD-10).

Supplementary Fig. S1 presents the flowchart of the final study episodes (n=636,543 episodes from n=413,435 patients). We included psychiatric sickness absence episodes with the most common diagnoses in working-age populations and excluded the rare diagnoses in this population, such as schizophrenia and neurodegenerative disorders. The included diagnostic groups were as follows: unipolar depressive episode (F32, F33), anxiety disorders, (i.e., neurotic, stress-related and somatoform disorders; F4), non-organic sleep disorders (F51), psychoactive substance use disorders (F1); bipolar disorder,mixed/unspecified (F31.6-F31.9, F31); manic episodes of bipolar disorder (F30, F31.0-F31.2), and depressive episodes of bipolar disorder (F31.3-F31.5). To examine the severity of the depressive disorders, we further divided unipolar depressive disorder into four sub-groups: mild (F32.0, F33.0), moderate (F32.1, F33.1), severe without psychotic symptoms (F32.2, F33.2), and severe with psychotic symptoms (F32.3, F33.3) when this information was available. Nested and successive episodes (when the gap between two episodes was shorter than two days) were considered a single episode. We applied a hierarchy rule for diagnoses if the diagnoses for the merged episodes differed. As previously, the diagnosis at the highest level of the hierarchy was chosen as the primary diagnosis of the merged episode (Törmälehto *et al*., 2022). Unipolar depressive episodes were denoted as bipolar depressive episodes if there was any history of unipolar manic or bipolar disorder in the register.

### Photoperiods

We estimated the photoperiod (hours of daylight) for each calendar day by subtracting the time of sunset from the time of sunrise in Helsinki (60°N), Finland. The 71-day photoperiods were based on the astronomical seasons; the summer and winter solstices (June 21 and December 21 or 22) and the vernal and autumnal equinoxes (March 20 or 21 and September 22 or 23), daylength, and the pace of change in daylength, as previously (Törmälehto *et al*., 2022) (see also Supplementary Table S1). Also, we defined the sub-photoperiods as follows: a slowly increasing (winter solstice +70 days), a rapidly increasing (end of slowly increasing +70 days), a slowly decreasing (summer solstice +70 days), a rapidly decreasing (end of slowly decreasing +70 days) photoperiod (see photoperiods illustrated in a figure nested in Table 1). For a sensitivity analysis, we further split the rapidly increasing and rapidly decreasing photoperiods into a beginning (30 days) and a tail end (40 days) of the photoperiod.

### Statistical analysis

Based on the first day of the sickness absence episode, we counted daily counts of new sickness absence episodes between January 1, 2006, and December 31, 2017. These types of data are subject to bias, as several factors other than seasonality may affect sickness absence during different seasons, such as weather events, holidays, vacations, and access to healthcare (Zhang *et al*., 2021). Therefore, the daily counts were standardized into an index by dividing them by the annual daily averages (Törmälehto *et al*., 2022). We then counted the daily counts of each psychiatric diagnosis group and considered these daily numbers the ‘observed’ counts. The ‘expected’ counts were derived by multiplying the annual diagnosis-specific daily averages by the annual daily counts of all-cause sickness absence. In addition, we calculated the ratio of observed and expected (O/E) daily counts, which represents the adjusted risk of sickness absence on a specific calendar day due to a specific mental disorder. Finally, to adjust for trends in sickness absence rates, we aggregated all daily O/E values across the study period from 2006 to 2017 by calculating a weighted arithmetic mean of O/E for each diagnosis group and each calendar day from 1 to 365/366. The daily O/E was assigned a weight that took into account the share of the diagnosis-related sickness absences relative to all sickness absences due to mental disorders in a particular year. We described examples of O/E calculation and weighing in detail in our previous study on hospital admissions (Törmälehto *et al*., 2022).

The daily occurrence of new sickness absence episodes was inspected both as the O/E values and Z-scores. The Z-score describes the number of standard deviations by which the O/E value deviates from the mean O/E value. Using the Z-score we were able to standardize the distributions of each diagnosis group and enable their comparison even though the mean O/E values and standard deviations were different. Finally, we illustrated the seasonal variation against the calendar days and applied the method of locally estimated scatterplot smoothing (LOESS) to fit the curve. An identical smoothing parameter (0.1) was selected for all diagnosis groups to enable comparison. The selected smoothing parameter roughly reflects a period of one month (10% of 366 days). To determine whether the observed counts deviated from the expected counts during each photoperiod, we specified the mean O/E or Z-score with 95% confidence intervals across each photoperiod and each diagnosis group.

To examine the robustness of our findings, we analyzed the data by including only the first sickness absence period for each individual in the analytical dataset. We also excluded all patients with any psychiatric sickness absence within a two-year clearance period, 2004–2005 (resulting in n=409,574 episodes). The data preprocessing was carried out in SAS 9.4 and R 3.6.1 and the statistical analyses in SAS 9.4.

## Results

Of the total of 636,543 episodes during the follow-up (2006–2017), slightly more than half (55.6%) were due to unipolar depressive disorder, followed by anxiety disorders (33.6%) (Supplementary Table S2). Other diagnostic groups constituted 0.5% to 5.2% of the sickness absence episodes.

**Fig 1:**
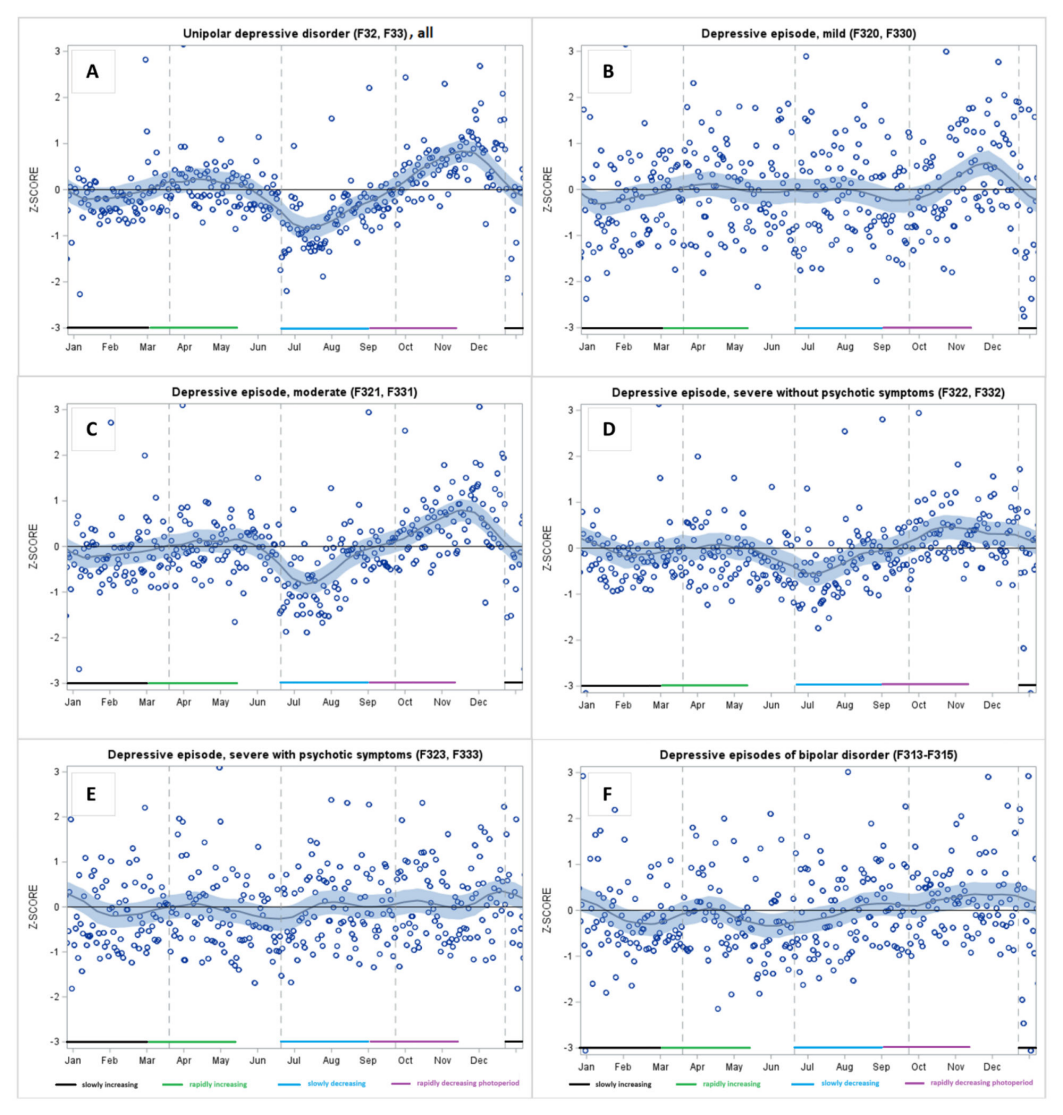
Smoothed LOESS time series of daily-based adjusted ratio of observed and expected diagnosis-specific sickness absence, expressed as standard Z-scores for unipolar depressive disorders, all (A), mild episodes (B), moderate episodes (C), severe episodes without psychotic symptoms (D), severe episodes with psychotic symptoms (E), and depressive episodes of bipolar disorder (F)

**Fig 2:**
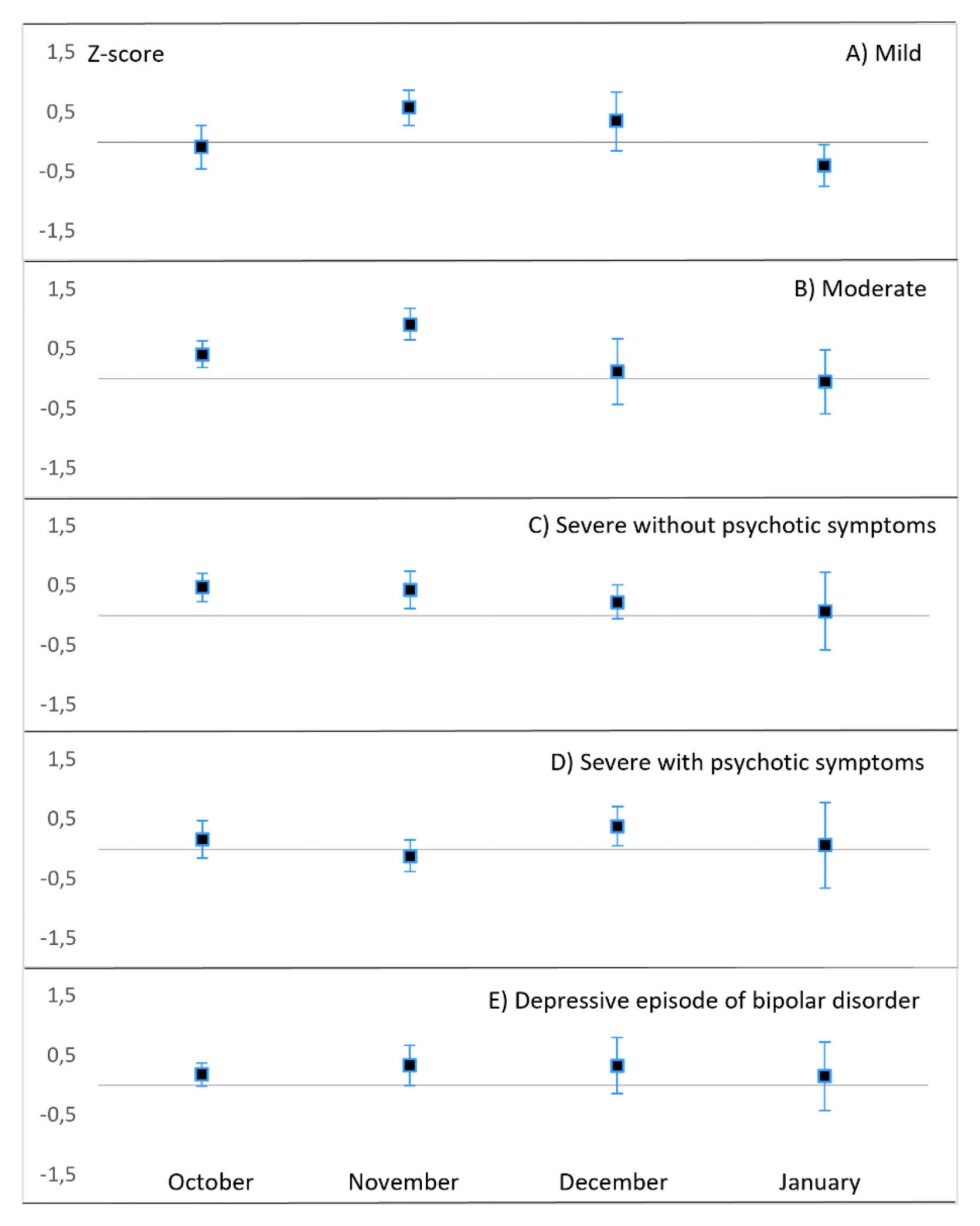
Adjusted ratio of observed and expected diagnosis-specific sickness absence, expressed as mean standard Z-scores (95% Cis) for unipolar depressive disorders (A) mild episodes; B) moderate episodes; C) severe episodes without psychotic symptoms; D) severe episodes with psychotic symptoms); and E) depressive episodes of bipolar disorder, each month from October to January

During a rapidly decreasing photoperiod (September to early November), we found a peak in sickness absence due to depressive disorders, specifically for moderate, severe without psychotic symptoms, and depressive episodes of bipolar disorder, but not for mild depression or severe depression with psychotic symptoms (Table 1 and Fig. 1). As further specified in Table 2, the exact timing of the peak was towards the end of the rapidly decreasing photoperiod (November). To deepen our knowledge of autumn-winter depression, we further examined whether there were significant peaks during the short photoperiod (November to January; for comparison, also October) that were not included in the initially specified rapidly decreasing photoperiods (Fig. 2). We found that mild episodes peaked in November, moderate and severe episodes without psychotic symptoms peaked in both October and November, bipolar depression peaked in November, and severe depression with psychotic symptoms peaked in December. Thus, there was a peak in late autumn–early winter in all forms of depressive disorders.

There was also a small peak in (overall) unipolar depression during the rapidly increasing photoperiod (from March to early May; Table 1 and Fig. 1). When we divided it into beginning and end phases, we were unable to specify which phase of the photoperiod was the dominant one in this peak (Table 2). Regarding the dips in depressive episodes, we found that the rates were lower than expected during the slowly decreasing photoperiod (from late June to the end of August) in the case of moderate disorders and severe disorders without psychotic symptoms, but not in the case of mild disorders, severe disorders with psychotic symptoms, or depressive episodes of bipolar disorder (Table 1 and Fig. 1).

**Fig 3:**
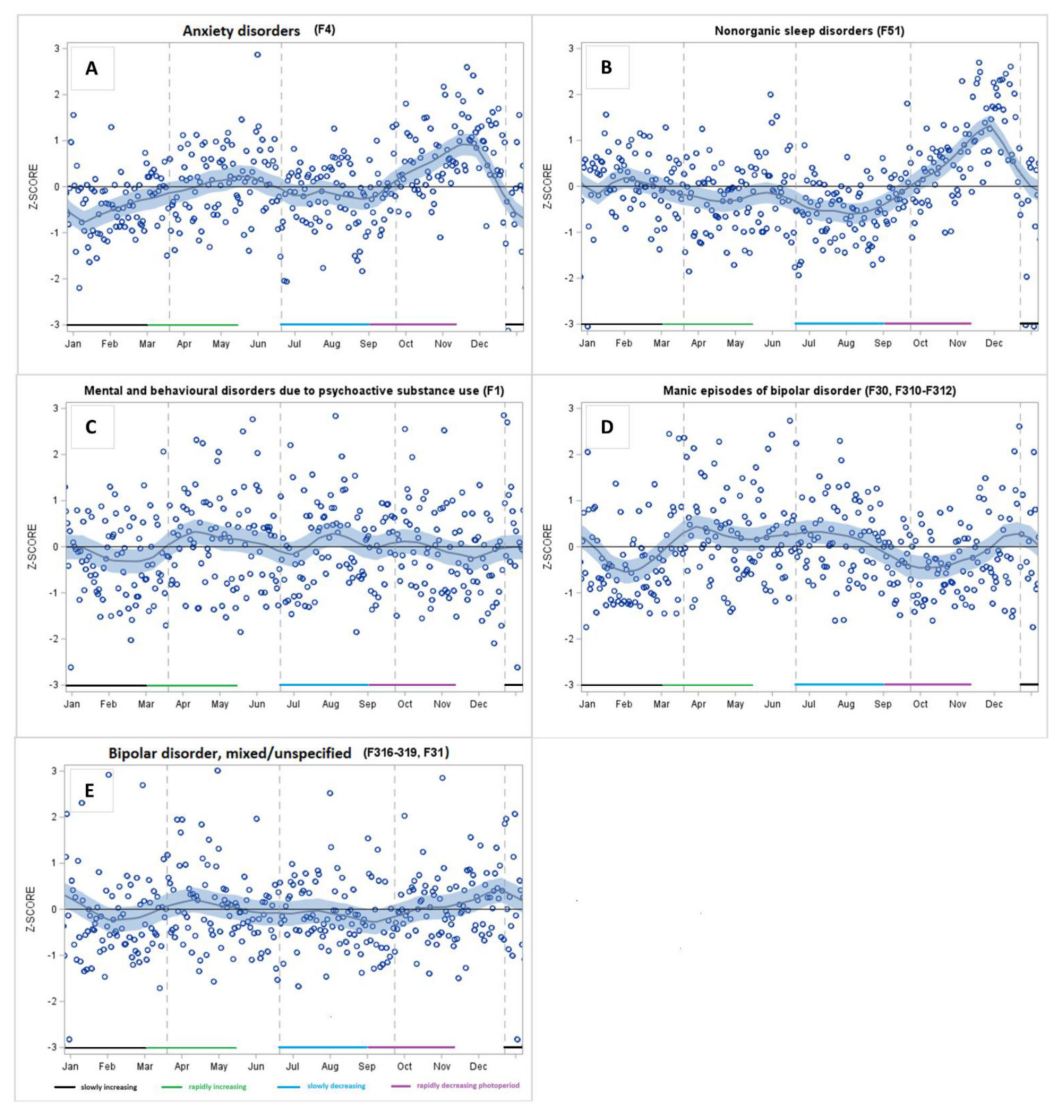
Smoothed LOESS time series of daily-based adjusted ratio of observed and expected diagnosis-specific sickness absence, expressed as standard Z-s cores for anxiety disorders (A), non-organic sleep disorders (B), psychoactive e substance use disorders (C), manic episodes of bipolar disorder (D), and mixed/unspecified episodes of bipolar disorder (E)

Regarding anxiety disorders, there was a peak in the autumn during the rapidly decreasing photoperiod (Table 1 and Fig. 3). Anxiety was at its lowest after the summer solstice, during the slowly decreasing photoperiod. It also fell considerably in December, which resulted in lower rates than expected in January to February (slowly increasing photoperiod). In the case of non-organic sleep disorders, we observed lower rates than expected in the spring (rapidly increased photoperiod), which remained over the summer period until the autumn, when they peaked. There was no significant seasonality in sickness absence due to substance use disorders (Table 1 and Fig. 3). We found similar results when we restricted the data to sickness absence due to alcohol use disorders (data not shown).

Manic episodes peaked during a rapidly increasing photoperiod (March–May) which continued across the slowly decreasing photoperiod, until late August (Table 1 and Fig. 3). We observed dips during a rapidly decreasing photoperiod (September to November) and during a slowly increasing photoperiod (January to February), and a small peak in December. For mixed/non-specified episodes of bipolar disorder, we found that sickness absence rates were lower than expected at the beginning of a rapidly decreasing photoperiod (September) (Table 2) and there was a peak in December (Fig. 3).

Supplementary Fig. S1 and Supplementary Fig. S2 present the results of the sensitivity analyses, with the LOESS figures showing non-standardized O/E ratio. Supplementary Table S3 and Supplementary Table S4 show the results from the analyses replicated with the outcomes including the first sickness absence episode only. These sensitivity analyses suggested no major differences to the original findings.

## Discussion

In this population-based study of 636,543 psychiatric sickness absence episodes over a total of 12 years, we found seasonal patterns in sickness absence due to several mental disorders. During a rapidly decreasing daylength from September to November, absenteeism due to common mental disorders, including depressive, anxiety, and non-organic sleep disorders peaked, whereas absenteeism due to manic episodes were at their lowest. For common mental disorders, the dips typically occurred in the spring (sleep) or the summer (most of the common mental disorders), whereas for manic episodes, rapidlyincreasing daylength in the spring was associated with elevated absence rates, which stayed at the same level from spring to summer. We did not observe the non-existence of seasonal variation in milder forms of clinical depressive disorders.

We found a general seasonal pattern for sickness absence due to unipolar depressive disorder, showing a peak at the end of the rapidly decreasing light, November, which changed in January. We observed lower absence rates than expected after the summer solstice, which continued from July to September. These findings corroborate previous research findings that have shown that clinically defined depressive disorders (Patten *et al.;* 2017, Overland *et al*., 2019), hospital admission due to depressive disorders (Dominiak *et al*., 2015; Fellinger *et al*., 2022; Törmälehto *et al*., 2022), and initiation of antidepressant medication (Gardarsdottir *et al*., 2010) follow a seasonal pattern. However, according to a review, several studies have also failed to show such seasonal patterns (Overland *et al*., 2019). Our findings were also similar to those regarding depression-related hospitalizations in Finland (Törmälehto *et al*., 2022). Along with the rapidly increasing photoperiod, we found a peak in the overall diagnosis of unipolar depression in April. This overall finding of a spring peak is in line with the results of previous studies of hospital admissions (Dominiak *et al*., 2015; Fellinger *et al*., 2022; Törmälehto *et al*., 2022) and suicides (Yu *et al*., 2020). The explanation for this paradox may be that the abruptly and rapidly increasing light exposure after the cloudy and dark period of the year, especially at northern latitudes, acts as a trigger for depressive feelings, which deepen among those who are already depressed (Partonen *et al*., 2004), whereas longer-term exposure to light during the summer enhances mood in general (Papadopoulos *et al*., 2005; Vyssoki *et al*., 2014). It seems that in the depressed, physiological responses in the brain and skin induced by exposure to light might be to varying extent abnormal when compared with those in healthy individuals (Partonen, 2015). Experimental studies have discovered potential pathways and therein mechanisms of action for these responses (LeGates *et al*., 2012; LeGates *et al*., 2014; Fernandez *et al*., 2018; Huang *et al*., 2019). Most of these studies have focused on the non-image forming effects of daylight which visible radiation induces through the eyes and are projected in the brain (Lazzerini Ospri *et al*., 2017). Here, it is of note that ultraviolet B radiation (Toledo *et al*., 2019) as well as infrared A radiation (Janssen *et al*., 2016) might also influence mood through the effects which they induce primarily in the skin there to be transmitted to the brain (Morrison and Nakamura, 2011; Ryu *et al*., 2015, Lowry *et al*., 2018).

We examined whether the seasonal pattern of sickness absence due to depressive disorders was similar at all levels of severity, as previous studies have suggested a winter peak more often in more severe depressive episodes (Overland *et al*., 2019). We found that moderate depression most consistently followed the general seasonal pattern with a peak in the autumn and a dip in the summer. However, our detailed analysis revealed a peak in all severity levels during at least one of the autumn months; thus, within clinical depression, the hypothesis was not supported.

Anxiety disorders peaked in November and dipped from July to August. However, there was also a clear drop in December, followed by sickness absence rates from January to February to be lower than expected. The diagnostic category of anxiety disorders (ICD-10 codes F40 to F48) includes a wide range of disorders, such as phobias, generalized anxiety disorder, adjustment disorder, and post-traumatic stress disorder. Self-reported seasonality has previously been associated with having panic disorder, agoraphobia, generalized anxiety disorder, or social phobia (Palmu *et al*., 2022) as well as self-reported anxiety symptoms (Oyane *et al*., 2008, Stordal *et al*., 2008) although null findings have also been reported (Winthorst *et al*., 2011, Friborg *et al*., 2012). To our knowledge, only one study has used register data on healthcare use (Zhang *et al*., 2021), and the findings were in line with our results although the seasonal patterns were only observed before adjusting for total number of healthcare appointments.

We found that sickness absence due to non-organic sleep disorders was similarly elevated in the winter period, as were depressive and anxiety disorders. This is in accordance with previous findings that sleep problems increase during the darkest winter period (Friborg *et al*., 2012; Kripke *et al*., 2015 [updated 2022]; Sivertsen *et al*., 2021), also in extreme photoperiodic conditions, such as in Antarctica (Mairesse *et al*., 2019). Our findings fit well with the definition of midwinter insomnia found in the highest latitude areas. Midwinter insomnia may vary from moderate difficulties falling asleep to nearly total insomnia throughout the night (Johnsen *et al*., 2012). However, these findings may also reflect sleep disturbances associated with SAD, such as excessive sleeping, and daytime tiredness during the winter period (Rosenthal *et al*., 1984). This is a plausible explanation, as we found a dip in absence due to sleep disorders from April to the end of the summer period. A study in Norway reported similar reduced sleep problems in spring (Johnsen *et al*., 2012) although opposite findings have also been reported (Putilov, 2017). However, some studies have suggested that the association between light exposure and sleep may also be dependent on ambient (outdoor) temperatures (Putilov, 2017, Min *et al*., 2021).

We found no significant seasonality in sickness absence due to substance use disorders, although the low incidence (1.2% of all episodes) suggests underrepresentation of these disorders in the data. A previous study reported a peak in substance use disorder-related healthcare appointments during the autumn and a decrease during the summer although this pattern did not hold after adjustment for seasonal variation in all-cause visits (Zhang *et al*., 2021). Self-reported seasonality has also been associated with clinically defined alcohol use disorders (Palmu *et al*., 2022), and thus, more research is needed to increase knowledge on seasonality in substance use disorders.

Our findings support the consensus on a seasonal pattern in bipolar disorder (Geoffroy *et al*., 2014; Kripke *et al*., 2015 [updated 2022]; Parker *et al*., 2017; Rosenthal *et al*., 2021). We found a peak in absenteeism due to manic episodes during the spring (under the rapidly increasing photoperiod) as well as in the summer (under the long and slowly decreasing photoperiods), and lower rates than expected throughout the autumn (under the rapidly decreasing photoperiod), with a slight increase again in January (under the slowly increasing photoperiod). Our findings regarding manic episodes fit relatively well with the hypothesis of rapidly changing light exposures triggering or preventing manic episodes (Rosenthal *et al*., 2020; Rosenthal *et al*., 2021). A mechanism of action for this hypothesis involves the rapidly increasing light exposure stimulating the pineal gland, resulting in reduced or suppressed melatonin secretion, which in turn triggers a manic episode (Lewy *et al*., 1981). A trait of hypersensitivity to light in bipolar disorder that induces not only greater than normal suppression of melatonin secretion by light, but also lower than normal melatonin concentrations at night as well as in the morning (Beck-Friis *et al*., 1984), might explain our finding that sickness absence due to a manic episode was most frequent during the period extending from the rapidly increasing photoperiod (March) to the slowly decreasing photoperiod (August) when the mornings are still relatively bright. Other than melatonin related hypotheses comprise of neurotransmitters, such as serotonin (Rosenthal *et al*., 2020) and neurotrophic factors (Frey *et al*., 2013) as well as sleep deprivation disrupting the circadian rhythms among susceptible individuals (Bunney and Bunney, 2013). However, the mechanisms are still not well understood in detail.

Mixed episodes of bipolar disorder include mood switches that cycle rapidly, more frequent psychotic symptoms, high levels of comorbidities and substance use disorders, a poorer prognosis, and a high suicide risk (Grunze *et al*., 2018). There was no significant seasonality in this diagnostic group, except a dip in the early autumn. A previous study suggested that mixed episodes peak in early spring as well as in the middle of or in the late summer (Geoffroy *et al*., 2014). It is possible that mixed episodes with more severe features are less affected by seasonal light exposures than other types of bipolar disorder. However, in our study, this group also included non-specified bipolar disorder (coded as either F31, F31.8, or F31.9), thus, this patient group is likely to be heterogeneous.

The strengths of our study include its extensive, population-based register data on a wide variety of psychiatric sickness absence claims covering a total of 12 years of sickness absence. The geographical location of Finland at high latitudes is ideal for studies on seasonal variation in mental disorders because of extreme seasonal variation in daylength. In the analyses, we carefully controlled for administrative and external fluctuation in sickness absence due to other factors than seasonality, such as holidays, weather, and access to care effects. The study has several limitations. With an ecological correlational study, any assumptions of causality are precluded. Furthermore, sickness absence data do not capture day-to-day variation in symptomatology, and there may have been a delay in help-seeking after the onset of symptoms. Only diagnosed cases with sickness absence are in the sickness absence records, thus, undiagnosed and untreated cases as well as those treated without sickness absence were not included in our study. Comorbid conditions are also common although the dataset only included the main diagnosis. For example, if anxiety disorder is the main diagnosis and the person also has depression, only anxiety disorder is recorded as a cause of sickness absence.

The exposure in our study was photoperiod (daylength), which we used as a proxy for daily-based solar insolation. The findings from the country near the equator (Ghana) suggested that seasonal variation in sleep and mood was small or non-existing, which gives some support for the usefulness of photoperiod as an exposure (Friborg *et al*., 2012). However, variation in daily exposure to solar radiation has been observed in geographical areas located at the same latitudes (Rosenthal *et al*., 2021). This variation is caused by other weather-related conditions that vary year by year, such as cloudiness and precipitation. In the future, studies could use more detailed meteorological measures of light exposure. Moreover, future studies should address the issue of temperature in combination with light exposure. Potential mechanisms, such as behavioral, hormonal, and neurotropic factors, should be addressed in more detail (Molendijk *et al*., 2012; Frey *et al*., 2013; Overland *et al*., 2019; Rosenthal *et al*., 2020).

The findings of this study are probably applicable to a high-latitude country with similar sickness absence practices as in Finland. In Finland, people are eligible to sickness absence allowances irrespective of their employment status. However, those who are severely ill or disabled from childhood are usually granted a work disability pension at the age of 16. They are not included in our study.

Finally, we have only discussed possible bioclimatic explanations for the observed associations. Even though we adjusted our analyses for administrative and external variation in sickness absence, there may still be psychosocial or cultural factors that partly explain the peaks and dips in sickness absence. For example, working life may follow a ‘psychosocial cycle’ in which a new start after each summer brings excessive expectations of employee productivity and innovation, and an increased workload which piles up towards the holiday season in the end of the year.

In sum, the results of this population-based register linkage study from a high-latitude country, Finland, suggest seasonal variation in sickness absence due to common mental disorders (depressive, anxiety, and non-organic sleep disorders), and bipolar disorder. The seasonality in sickness absence due to depressive disorders does not seem to be affected by disease severity. Our findings support the hypothesized role of rapid changes in light exposure in bipolar disorder, as both a triggering and protective factor although other contributing factors may exist. The findings of this study add to the evidence of seasonality in mental disorders. However, as extreme heatwaves are increasingly common in many countries during summer, the patterns in mental health-related outcomes may change (Ebi *et al*., 2021). The present results may also contribute to precision medicine (Le-Niculescu *et al*., 2021), and can inform clinicians and workplaces to prepare for varying healthcare needs.

## Supplementary Material

Supplementary material

## Figures and Tables

**Table 1:** Mean standardized Z-scores of the observed/expected count (with 95% confidence intervals) of new diagnosis-specific sickness absence periods 2006-2017 by changing seasonal photoperiods

	Seasonal photoperiods, later phase of the year		Seasonal photoperiods and their timing over the year	Seasonal photoperiods, earlier phase of the year	
ICD-10 diagnosis for sickness absence	Slowly decreasing light	Rapidly decreasing light		Slowly increasing light	Rapidly increasing light
Unipolar depressive disorder, all	-0.71 (-0.85- -0.57)	0.29 (0.11-0.47)	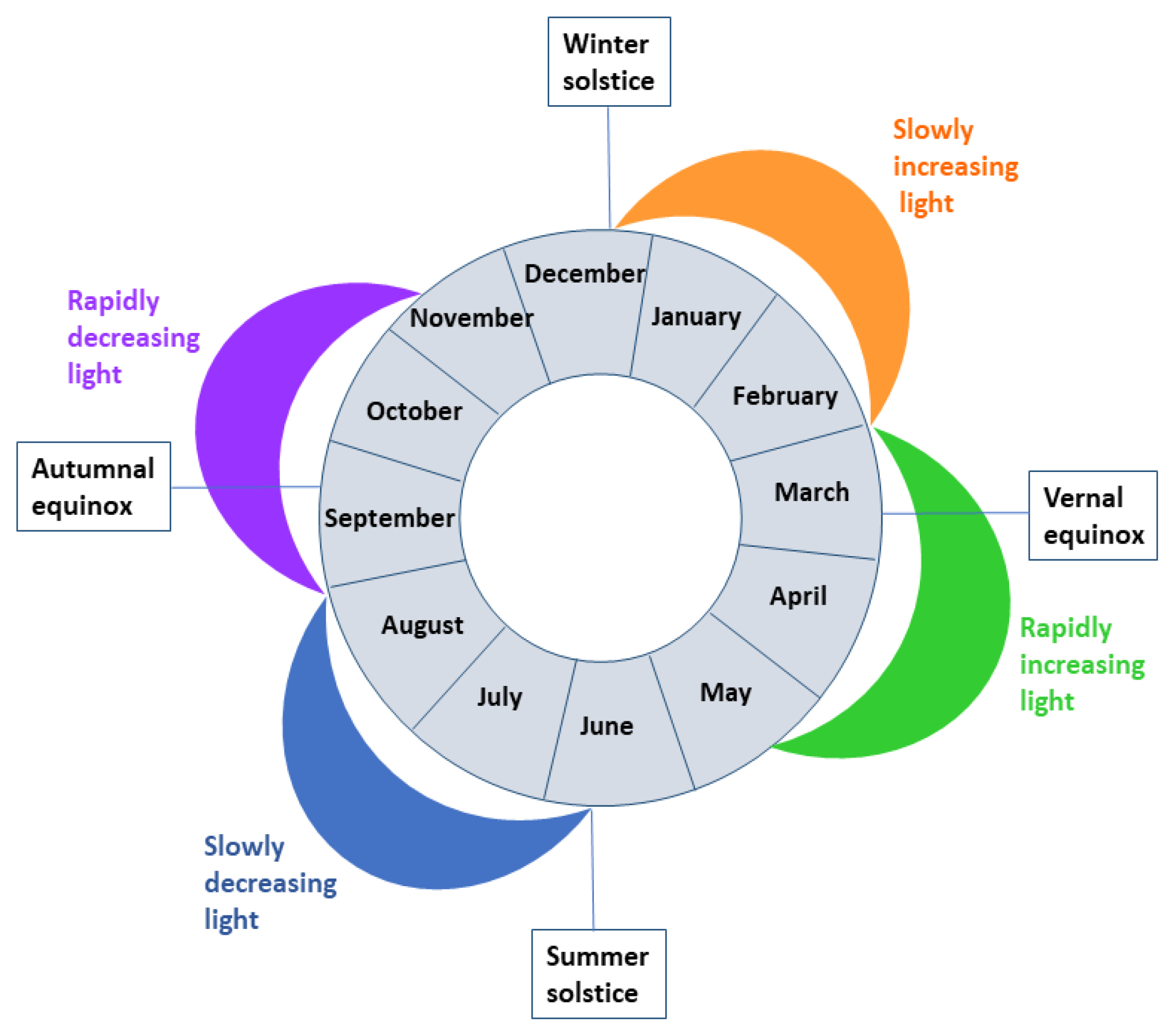	-0.18 (-0.49-0.14)	0.19 (0.03-0.35)
Mild	-0.06 (-0.29-0.17)	-0.05 (-0.28-0.17)		-0.24 (-0.50-0.02)	0.07 (-0.15-0.28)
Moderate	-0.61 (-0.78- -0.45)	0.32 (0.15-0.50)		-0.23 (-0.53-0.08)	0.09 (-0.10-0.27)
Severe	-0.38 (-0.54- -0.22)	0.29 (0.10-0.49)		0.03 (-0.31-0.36)	0.04 (-0.20-0.27)
Severe with psychotic symptoms	-0.03 (-0.23-0.17)	0.06 (-0.14-0.27)		0.03 (-0.31-0.36)	0.00 (-0.20-0.20)
Depressive episodes of bipolar disorder	-0.05 (-0.26-0.17)	0.19 (0.02-0.37)		-0.02 (-0.33-0.29)	-0.04 (-0.26-0.19)
Anxiety disorders	-0.25 (-0.42- -0.08)	0.28 (0.11-0.45)		-0.62 (-0.87- -0.37)	0.02 (-0.14-0.17)
Non-organic sleep disorders	-0.61 (-0.75- -0.47)	0.22 (0.06-0.38)		-0.12 (-0.37-0.14)	-0.24 (-0.40- -0.09)
Substance use disorders	0.10 (-0.13-0.32)	0.04 (-0.18-0.25)		-0.07 (-0.31-0.16)	0.13 (-0.13-0.38)
Bipolar disorder, mixed/non-specified	-0.11 (-0.27-0.05)	-0.05 (-0.23-0.14)		0.04 (-0.32-0.41)	0.11 (-0.10-0.32)
Manic episodes of bipolar disorder	0.27 (0.05-0.49)	-0.42 (-0.57- -0.28)		-0.28 (-0.52- -0.04)	0.27 (0.02-0.52)

*Note.* The photoperiods were defined using information on astronomical seasons (summer and winter solstices and vernal and autumnal equinoxes), daylength and pace of change in daylength (these were based on sunrise and sunset times in Helsinki (60°N), Finland; see also Törmälehto et al (2022). Values above 0 denote higher than expected sickness absence rates and values below 0 denote lower than expected sickness absence rates.

**Table 2:** Mean standardized Z-scores for observed to expected (O/E) count (with 95% confidence intervals) of new diagnosis-specific sickness absence periods 2006–2017 by the phases of rapidly changing seasonal photoperiods

	Rapidly decreasing light		Rapidly increasing light	
Diagnostic groups of sickness absence episodes	Beginning phase	End phase	Beginning phase	End phase
Unipolar depressive disorder, all	-0.18 (-0.35- -0.01)	0.61 (0.37-0.85)	0.17 (-0.08-0.43)	0.21 (-0.01-0.43)
Mild	-0.30 (-0.58- -0.02)	0.10 (-0.22-0.42)	0.08 (-0.19-0.36)	0.07 (-0.22-0.35)
Moderate	0.00 (-0.19-0.20)	0.56 (0.32-0.80)	0.04 (-0.21-0.29)	0.14 (-0.10-0.39)
Severe	-0.03 (-0.24-0.17)	0.50 (0.22-0.78)	0.05 (-0.25-0.34)	0.08 (-0.28-0.45)
Severe with psychotic symptoms	0.05 (-0.23-0.32)	0.07 (-0.21-0.35)	0.01 (-0.26-0.28)	-0.03 (-0.31-0.26)
Depressive episodes of bipolar disorder	0.17 (-0.11-0.45)	0.26 (0.05-0.46)	-0.12 (-0.36-0.11)	0.01 (-0.34-0.36)
Anxiety disorders	-0.16 (-0.38-0.06)	0.51 (0.28-0.74)	-0.20 (-0.39-0.00)	0.13 (-0.08-0.35)
Non-organic sleep disorders	-0.24 (-0.46- -0.03)	0.43 (0.22-0.65)	-0.20 (-0.42-0.02)	-0.33 (-0.53- -0.12)
Substance use disorders	-0.07 (-0.30-0.16)	0.12 (-0.21-0.45)	-0.13 (-0.47-0.22)	0.30 (-0.02-0.62)
Bipolar disorder, mixed/non-specified	-0.32 (-0.55- -0.10)	0.12 (-0.13-0.37)	0.01 (-0.28-0.30)	0.17 (-0.13-0.47)
Manic episodes of bipolar disorder	-0.32 (-0.53- -0.11)	-0.42 (-0.62- -0.22)	0.11 (-0.25-0.47)	0.29 (-0.04-0.61)

*Note.* Values above 0 denote higher than expected sickness absence rates and values below 0 denote lower than expected sickness absence rates.

## Data Availability

These register-based data will not be publicly available. However, researchers may contact the first author (marianna.virtanen@uef.fi) or the sixth author (christian.hakulinen@helsinki.fi) for further information.
