# Supplementary material for: Seasonal patterns of sickness absence due to diagnosed mental disorders: a nationwide 12-year register linkage study
Source: Epidemiol Psychiatr Sci. Author manuscript; Available in PMC 2023 Nov 29. (PMC7615330; doi:10.1017/S2045796023000768)
Supplement: Supplementary material [file EMS189664-supplement-Supplementary_material.pdf]

Supplementary material

Contents **Page**

Fig. S1. Flowchart of selection of register data for analytical sample 30

Additional information about measures (Supplementary Table S1) 31

Additional results

Supplementary Table S2 32

Fig. S2 33

Fig. S3 34

Supplementary Table S3 35

Supplementary Table S4 36

Fig. S1. Flowchart of selection of register data for analytical sample

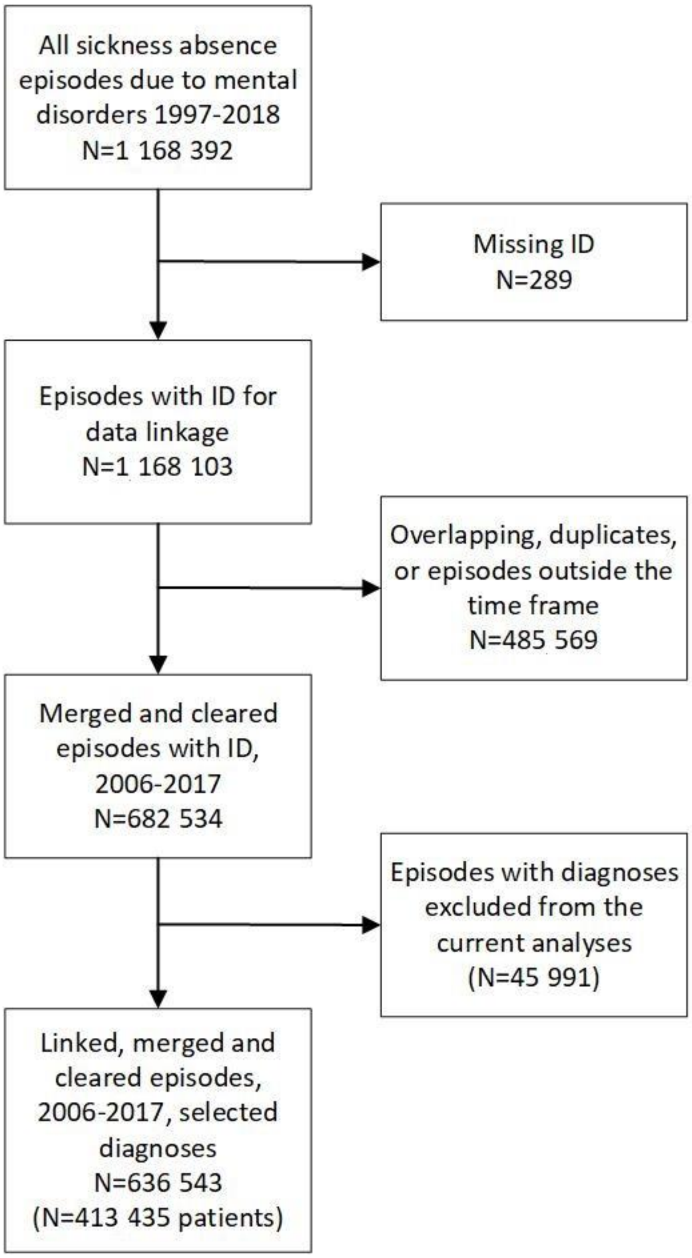

Supplementary Table S1. Photoperiods, starting and ending dates of the period, number of days in the period, mean daylength in hours (h) and mean pace of change in daylength in minutes (min) per day

**Photoperiod**[1]	**Starting date**	**Ending date**	**Days (n)**	**Daylength (h)**[2]	**Pace of change (min)**[2]
Slowly increasing	21 Dec	1 Mar	71	7.6	3.9
Rapidly increasing	2 Mar	11 May	71	13.7	5.4
Start of rapidly increasing	22 Feb	1 Apr	39	11.6	5.5
End of rapidly increasing	2 Apr	11 May	40	15.2	5.4
Slowly decreasing	21 Jun	30 Aug	71	17.1	–3.9
Rapidly decreasing	31 Aug	9 Nov	71	11.1	–5.4
Start of rapidly decreasing	23 Aug	30 Sep	39	13.3	–5.4
End of rapidly decreasing	1 Oct	9 Nov	40	9.7	–5.3

Notes: [1]The photoperiods were based on astronomical seasons (summer and winter solstices and vernal and autumnal equinoxes), daylength and pace of change in daylength; [2]Daylength and pace of change in daylength are based on the sunrise and the sunset times in Helsinki, Finland (60°N). The dates of astronomical seasons and sunrise and sunset times were derived from the University of Helsinki Almanac Office.

Supplementary Table S2. Number of sickness absence episodes in each mental disorder group

Diagnosis-specific group	n (%) of episodes
Unipolar depressive episode (F32, F33)	353,690 (55.6)
Anxiety disorders (F4)	213,842 (33.6)
Non-organic sleep disorders (F51)	33,357 (5.2)
Psychoactive substance use disorders (F1)	7,872 (1.2)
Bipolar disorder, mixed/non-specified (F316-319, F31)	15,279 (2.4)
Manic episodes of bipolar disorder (F30, F310-312)	3,199 (0.5)
Depressive episodes of bipolar disorder (F313-315)	9,304 (1.5)
Total	636,543 (100.0)

Fig. S2 Smoothed LOESS time series of daily-based ratio of observed and expected diagnosis-specific sickness absence, expressed as observed–expected ratio (O/E) for unipolar depressive disorders, all (A), mild episodes (B), moderate episodes (C), severe episodes without psychotic symptoms (D), severe episodes with psychotic symptoms (E), and depressive episodes of bipolar disorder (F)

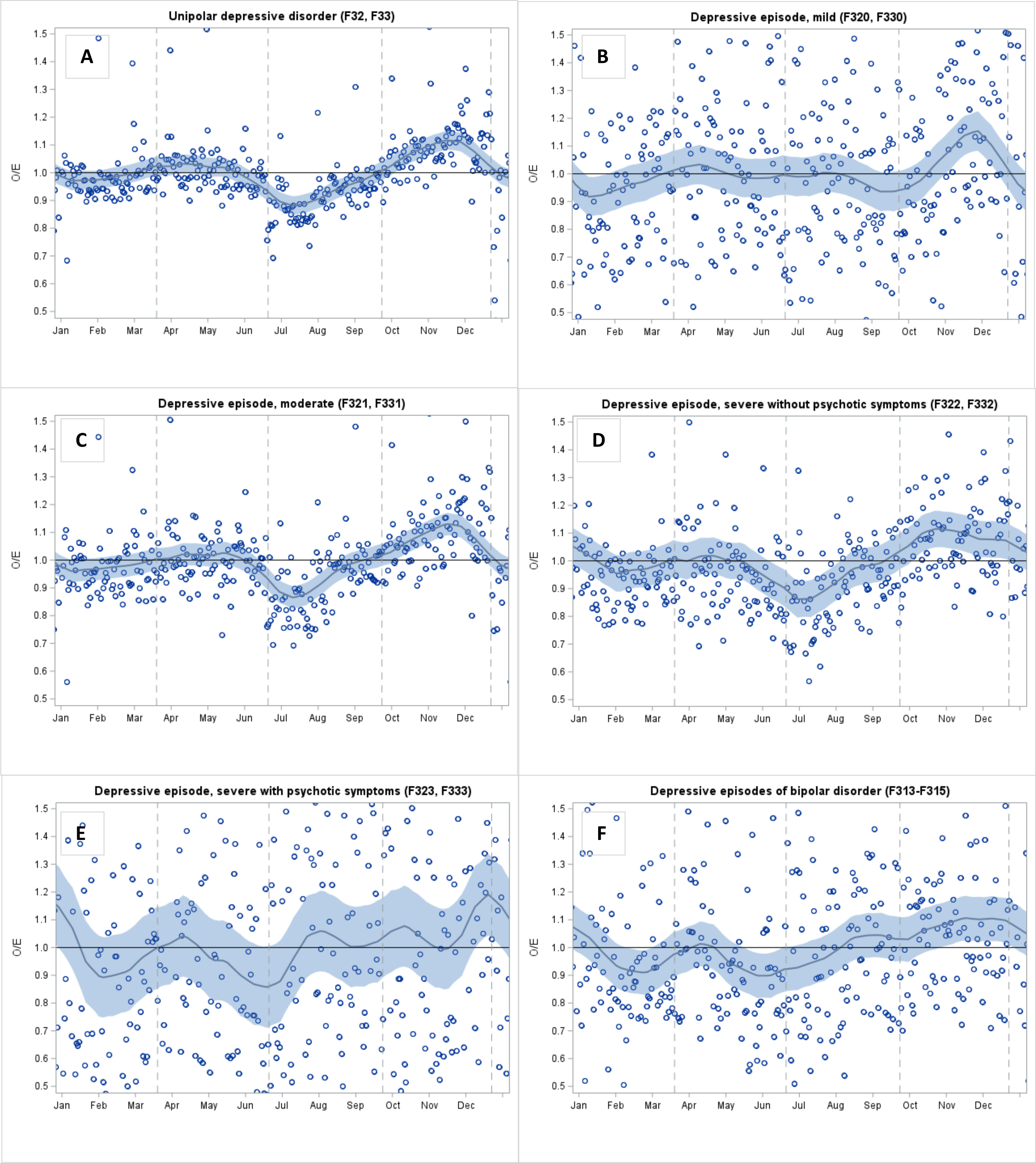

Fig. S3 Smoothed Loess time series of daily-based ratio of observed and expected diagnosis-specific sickness absence, expressed as the observed-expected ratio (O/E) for anxiety disorders (A), non-organic sleep disorders (B), psychoactive substance use disorders (C), manic episodes of bipolar disorder (D), and mixed/unspecified episodes of bipolar disorder (E)

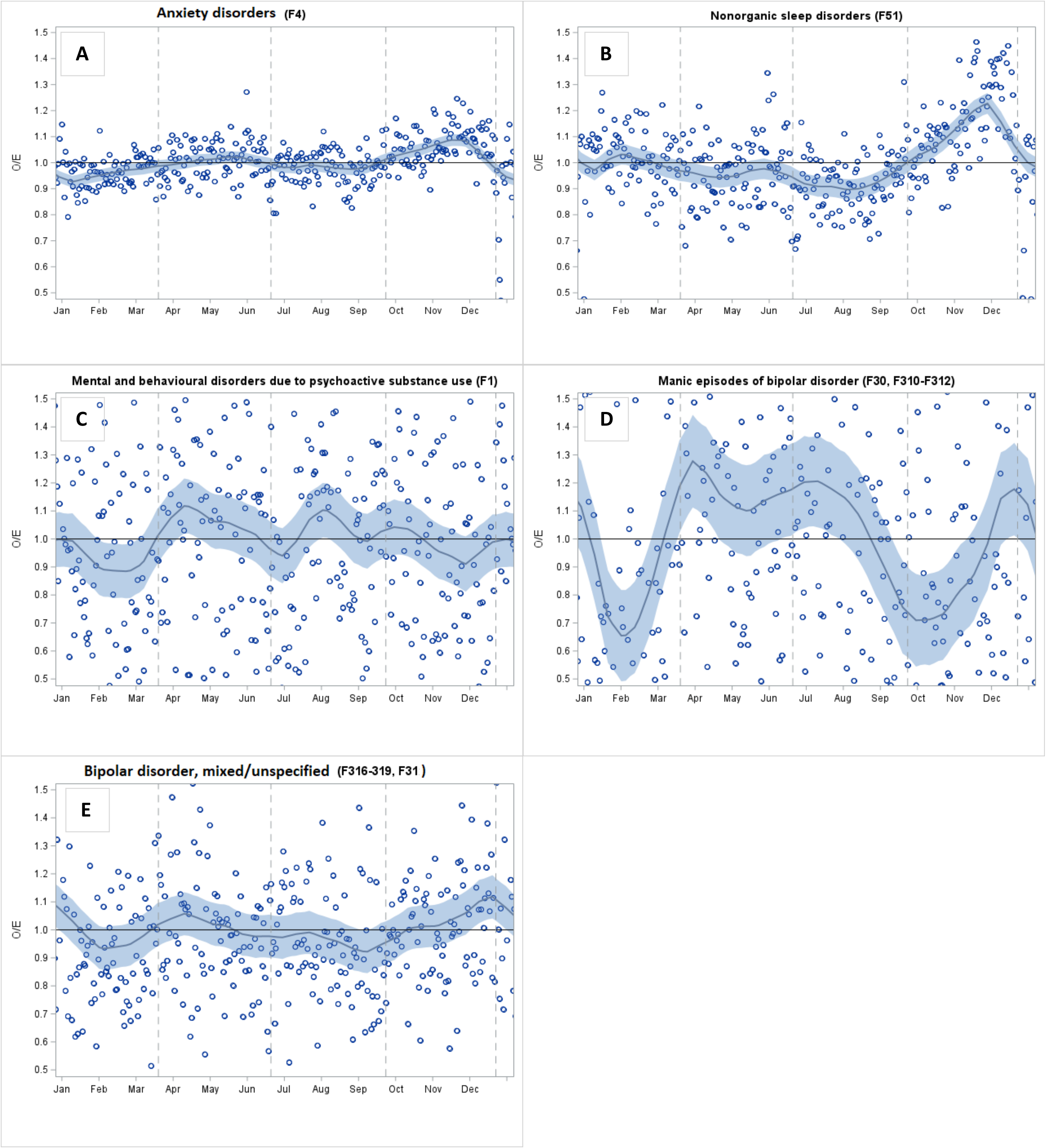

Supplementary Table S3. Mean standardized Z-scores for observed to expected (O/E) count (with 95% confidence intervals) of new diagnosis-specific sickness absence periods 2006–2017 (the first period only) by changing seasonal photoperiods

	**Seasonal photoperiods, later phase of the year**		**Seasonal photoperiods, earlier phase of the year**	
**ICD-10 diagnosis for sickness absence**	**Slowly decreasing light**	**Rapidly decreasing light**	**Slowly increasing light**	**Rapidly increasing light**
Unipolar depressive disorder	-0.84 (-0.97- -0.71)	0.20 (0.02-0.38)	-0.03 (-0.35-0.29)	0.38 (0.23-0.53)
Depressive episodes of bipolar disorder	-0.14 (-0.37-0.10)	0.00 (-0.20-0.21)	0.08 (-0.23-0.38)	0.16 (-0.06-0.39)
Anxiety disorders	-0.14 (-0.34-0.06)	0.15 (-0.03-0.33)	-0.54 (-0.78- -0.30)	0.12 (-0.07-0.30)
Non-organic sleep disorders	-0.58 (-0.73- -0.43)	0.21 (0.04-0.38)	-0.07 (-0.33-0.20)	-0.21 (-0.38- -0.03)
Substance use disorders	0.08 (-0.14-0.29)	0.07 (-0.14-0.27)	0.07 (-0.17-0.31)	0.10 (-0.16-0.36)
Bipolar disorder, mixed/non-specified	-0.11 (-0.32-0.09)	-0.15 (-0.35-0.04)	0.08 (-0.26-0.42)	0.23 (0.02-0.44)
Manic episodes of bipolar disorder	0.28 (0.03-0.54)	-0.34 (-0.52- -0.16)	-0.24 (-0.50-0.01)	0.21 (-0.02-0.45)

Note. Values above 0 denote higher than expected sickness absence rates and values below 0 denote lower than expected sickness absence rates.

Supplementary Table S4. Mean standardized Z-scores for observed to expected (O/E) count (with 95% confidence intervals) of new diagnosis-specific sickness absence periods (the first period only) 2006–2017 by rapidly changing seasonal photoperiods

	Rapidly decreasing light		Rapidly increasing light	
**ICD-10 diagnosis for sickness absence**	**Beginning phase**	**End phase**	**Beginning phase**	**End phase**
Unipolar depressive disorder	-0.33 (-0.51- -0.15)	0.54 (0.31-0.76)	0.38 (0.13-0.63)	0.33 (0.14-0.52)
Depressive episodes of bipolar disorder	-0.07 (-0.36-0.22)	0.03 (-0.22-0.29)	0.19 (-0.11-0.48)	0.10 (-0.22-0.42)
Anxiety disorders	-0.20 (-0.46-0.05)	0.30 (0.07-0.53)	-0.05 (-0.28-0.18)	0.20 (-0.06-0.45)
Non-organic sleep disorders	-0.30 (-0.53- -0.08)	0.48 (0.26-0.70)	-0.18 (-0.36-0.00)	-0.27 (-0.54-0.01)
Substance use disorders	0.01 (-0.24-0.25)	0.13 (-0.16-0.42)	-0.12 (-0.50-0.26)	0.29 (-0.01-0.59)
Bipolar disorder, mixed/non-specified	-0.35 (-0.57- -0.12)	-0.07 (-0.35-0.22)	0.07 (-0.21-0.34)	0.33 (0.03-0.64)
Manic episodes of bipolar disorder	-0.23 (-0.48-0.02)	-0.40 (-0.64- -0.16)	0.07 (-0.30-0.45)	0.22 (-0.04-0.48)

Note. Values above 0 denote higher than expected sickness absence rates and values below 0 denote lower than expected sickness absence rates.
