# Supplementary material for: Seasonal patterns of sickness absence due to diagnosed mental disorders: a nationwide 12-year register linkage study
Source: Epidemiol Psychiatr Sci. 2023 Nov 9;32:e64. doi: 10.1017/S2045796023000768 (PMC7615330; doi:10.1017/S2045796023000768)
Supplement: Supplementary file 1 [file S2045796023000768sup001.docx]

**Supplementary material**

**Contents** 	**Page**

Fig. S1. Flowchart of selection of register data for analytical sample		2

Additional information about measures (Supplementary Table S1)		3

Additional results					

	Supplementary Table S2				4

	Fig. S2					5

	Fig. S3					6

	Supplementary Table S3				7

	Supplementary Table S4				8

**Fig. S1.** Flowchart of selection of register data for analytical sample

**Supplementary Table S1**. Photoperiods, starting and ending dates of the period, number of days in the period, mean daylength in hours (h) and mean pace of change in daylength in minutes (min) per day

| **Photoperiod**^**1**^ | **Starting date** | **Ending date** | **Days (n)** | **Daylength (h)**^**2**^ | **Pace of change (min)**^**2**^ |
| --- | --- | --- | --- | --- | --- |
| Slowly increasing | 21 Dec | 1 Mar | 71 | 7.6 | 3.9 |
| Rapidly increasing | 2 Mar | 11 May | 71 | 13.7 | 5.4 |
| Start of rapidly increasing | 22 Feb | 1 Apr | 39 | 11.6 | 5.5 |
| End of rapidly increasing | 2 Apr | 11 May | 40 | 15.2 | 5.4 |
| Slowly decreasing | 21 Jun | 30 Aug | 71 | 17.1 | –3.9 |
| Rapidly decreasing | 31 Aug | 9 Nov | 71 | 11.1 | –5.4 |
| Start of rapidly decreasing | 23 Aug | 30 Sep | 39 | 13.3 | –5.4 |
| End of rapidly decreasing | 1 Oct | 9 Nov | 40 | 9.7 | –5.3 |

**Notes:**^ 1^The photoperiods were based on astronomical seasons (summer and winter solstices and vernal and autumnal equinoxes), daylength and pace of change in daylength; ^2^Daylength and pace of change in daylength are based on the sunrise and the sunset times in Helsinki, Finland (60°N). The dates of astronomical seasons and sunrise and sunset times were derived from the University of Helsinki Almanac Office.

**Supplementary Table S2.** Number of sickness absence episodes in each mental disorder group

| **Diagnosis-specific group** | **n (%) of episodes** |
| --- | --- |
| Unipolar depressive episode (F32, F33) | 353,690 (55.6) |
| Anxiety disorders (F4) | 213,842 (33.6) |
| Non-organic sleep disorders (F51) | 33,357 (5.2) |
| Psychoactive substance use disorders (F1) | 7,872 (1.2) |
| Bipolar disorder, mixed/non-specified (F316-319, F31) | 15,279 (2.4) |
| Manic episodes of bipolar disorder (F30, F310-312) | 3,199 (0.5) |
| Depressive episodes of bipolar disorder (F313-315) | 9,304 (1.5) |
| Total | 636,543 (100.0) |

				

**Fig. S2** Smoothed LOESS time series of daily-based ratio of observed and expected diagnosis-specific sickness absence, expressed as observed–expected ratio (O/E) for: (A) unipolar depressive disorders, all; (B) mild episodes; (C) moderate episodes; (D) severe episodes without psychotic symptoms; (E) severe episodes with psychotic symptoms; and (F) depressive episodes of bipolar disorder

**A**

**B**

**C**

**D**

**E**

**F**

                                                                                       

**Fig. S3** Smoothed Loess time series of daily-based ratio of observed and expected diagnosis-specific sickness absence, expressed as the observed-expected ratio (O/E) for: (A) anxiety disorders; (B) non-organic sleep disorders; (C) psychoactive substance use disorders; (D) manic episodes of bipolar disorder; and (E) mixed/unspecified episodes of bipolar disorder

**A**

**B**

**C**

**D**

**E**

**Supplementary Table S3.** Mean standardized Z-scores for observed to expected (O/E) count (with 95% confidence intervals) of new diagnosis-specific sickness absence periods 2006–2017 (the first period only) by changing seasonal photoperiods    

|  | **Seasonal photoperiods, later phase of the year** |  | **Seasonal photoperiods, earlier phase of the year** |  |
| --- | --- | --- | --- | --- |
| **ICD-10 diagnosis for sickness absence** | **Slowly decreasing light** | **Rapidly decreasing light** | **Slowly increasing light** | **Rapidly increasing light** |
| Unipolar depressive disorder | -0.84 (-0.97- -0.71) | 0.20 (0.02-0.38) | -0.03 (-0.35-0.29) | 0.38 (0.23-0.53) |
| Depressive episodes of bipolar disorder | -0.14 (-0.37-0.10) | 0.00 (-0.20-0.21) | 0.08 (-0.23-0.38) | 0.16 (-0.06-0.39) |
| Anxiety disorders | -0.14 (-0.34-0.06) | 0.15 (-0.03-0.33) | -0.54 (-0.78- -0.30) | 0.12 (-0.07-0.30) |
| Non-organic sleep disorders | -0.58 (-0.73- -0.43) | 0.21 (0.04-0.38) | -0.07 (-0.33-0.20) | -0.21 (-0.38- -0.03) |
| Substance use disorders | 0.08 (-0.14-0.29) | 0.07 (-0.14-0.27) | 0.07 (-0.17-0.31) | 0.10 (-0.16-0.36) |
| Bipolar disorder, mixed/non-specified | -0.11 (-0.32-0.09) | -0.15 (-0.35-0.04) | 0.08 (-0.26-0.42) | 0.23 (0.02-0.44) |
| Manic episodes of bipolar disorder | 0.28 (0.03-0.54) | -0.34 (-0.52- -0.16) | -0.24 (-0.50-0.01) | 0.21 (-0.02-0.45) |

*Note.* Values above 0 denote higher than expected sickness absence rates and values below 0 denote lower than expected sickness absence rates.

**Supplementary Table S4.** Mean standardized Z-scores for observed to expected (O/E) count (with 95% confidence intervals) of new diagnosis-specific sickness absence periods (the first period only) 2006–2017 by the phases of rapidly changing seasonal photoperiods

|  | **Rapidly decreasing light** |  | **Rapidly increasing light** |  |
| --- | --- | --- | --- | --- |
| **ICD-10 diagnosis for sickness absence** | **Beginning phase** | **End phase** | **Beginning phase** | **End phase** |
| Unipolar depressive disorder | -0.33 (-0.51- -0.15) | 0.54 (0.31-0.76) | 0.38 (0.13-0.63) | 0.33 (0.14-0.52) |
| Depressive episodes of bipolar disorder | -0.07 (-0.36-0.22) | 0.03 (-0.22-0.29) | 0.19 (-0.11-0.48) | 0.10 (-0.22-0.42) |
| Anxiety disorders | -0.20 (-0.46-0.05) | 0.30 (0.07-0.53) | -0.05 (-0.28-0.18) | 0.20 (-0.06-0.45) |
| Non-organic sleep disorders | -0.30 (-0.53- -0.08) | 0.48 (0.26-0.70) | -0.18 (-0.36-0.00) | -0.27 (-0.54-0.01) |
| Substance use disorders | 0.01 (-0.24-0.25) | 0.13 (-0.16-0.42) | -0.12 (-0.50-0.26) | 0.29 (-0.01-0.59) |
| Bipolar disorder, mixed/non-specified | -0.35 (-0.57- -0.12) | -0.07 (-0.35-0.22) | 0.07 (-0.21-0.34) | 0.33 (0.03-0.64) |
| Manic episodes of bipolar disorder | -0.23 (-0.48-0.02) | -0.40 (-0.64- -0.16) | 0.07 (-0.30-0.45) | 0.22 (-0.04-0.48) |

*Note.* Values above 0 denote higher than expected sickness absence rates and values below 0 denote lower than expected sickness absence rates.
